# Success in the Dental Profession

**Published:** 1863-01

**Authors:** C. C. Dills

**Affiliations:** Piqua, O.


### SUCCESS IN THE DENTAL PROFESSION.

BY C. C. DILLS, D. D. S.

### NO. 5.—GENIUS; HEALTH; ENERGY.

I'd have genius effective; and include
Health and energy, and a multitude
Of telling virtues, ne'er to be defined,
And yet existing in the thoughtful mind.
And I'd have genius more catholic than
In a thousand to be owned by one man;
I believe "what man has done, man may do,"
I believe genius is for all—for you.

To answer my purpose I must take issue with the classic acceptation of the term genius this far, that it is not a faculty so peculiarly *inborn* as one peculiarly *developed;* and therefore not one necessarily confined to the few, but accessible to the many. I will not deny but that there is here and there a natural bent of mind, strong and peculiar, and to which it is convenient and proper to apply the term genius; but I contend that this is its limited sense, and that another, broader, and as much called for, is that vitality, and effective working of character, producing the complete or extraordinery success. I would ever call that genius that *effected;* I would say that the only genius with which we have to do must necessarily accomplish,—must not dwell in the ideal, but become real,—become practical,—must succeed. I consider a complete success both the work of a genius, and worthy a genius. I would call success and genius synonymous terms, one including the other. What though I vulgarize the classics here, do I more than justice to the successful of life! Besides this is not the place to justify or elaborate my definition; I am writing on the very practical subject of success, and not on belles-lettres. I can simply find no better term to express those peculiar characteristics determining the successful man, than genius; if it is not such strictly speaking, it "yet borders nearly upon it, and very much at length runs up into it, and requires to be particularly considered with regard to things that look that way."

Now, while the intent of this article forbids a literary disquisition on the abstract terms of genius, it would yet be very proper that I should describe that peculiarity of character producing the extraordinary success, and which I term genius. But, unfortunately, herein does my idea of genius partake of the character of genius in the abstract,—it is indescribable.

The two grand prerogatives of genius are, indescribableness and rarity; these I would retain, as in the very nature of things necessary. For, could I describe genius, by all that is peculiar to it, it would cease to be such; and none the less so with my idea of it, for, the description would but serve as an *open-sesame* to this extraordinary success, which, of course, would cease to be extraordinary at all. But I have said that genius is for all; well, I did that under a poetic license; in prose I said, it was *accessible* to all,—so it is,—though all will not get it; "this is true, and that other is true." Do you still object? "What man has done, man may do." May he? Yes, he *may*,—but *will* he! No!—yet the chances that he will, and the certain benefit of the effort, are fully worth the trial. But not to trifle. I have already shown the propriety for using the term at all, and I will yet write as practically on it, as writers usually do on genius. And right here is another reason for adopting the term, that I may write in a manner somewhat desultory, may give "line upon line, precept upon precept, here a little, and there a little," as the case may call for. We may expect that it will be with some difficulty, and perhaps with some flounderings, that we even approximate an idea of those anomalous traits of character, which not only secure success, but secure the most complete, and with the most rapid and labor-saving strides : and we may well congratulate ourselves, in the end, for any knowledge of them we may have obtained. Now, I don't want to promise here in proportion to our desire, or probable anticipation : I have simply *asserted* in this article, as a reasonable fact, that a complete success is both the work of genius, and worthy a genius; that this genius is developmental, and accessible to all : I simply propose *doing* in this article what I can towards aiding a knowledge and development of this genius,—towards fostering some less regarded, and yet very potent elements of success in the dental profession.

First, then : Let the dentist who would have an effective genius, see to it that he has health and energy. For it is evident that the essence of this genius is vitality, and certainly the essence of vitality is health. And in the outset let me emphasize this fact, that nine-tenths of all the genius wanted, or to be acquired, or that is practical for us, is energy; and that nine-tenths of the remaining tenth is energy; and that the remaining decimal is scarcely to be distinguished from energy. Energy, vitality, life, *intensified*, is genius all told. I presuppose here, of course, the natural and inevitable *workings* of all these. And who can conceive of this state of things as resulting in any thing short of success. Then, repeat the thought over and over again in your mind,—think of it while awake, and dream of it while asleep, till it become so thoroughly infused into your whole being that you can but *act upon it*,—and the price of the suggestion may not be estimated short of the most complete success. Then will I not have written in vain, but will have shown the grand secret of success and genius to be, *the arousing and putting forth of all the energy, and faculties of the soul.*

"On the neck of the young man," says Hafiz, "sparkles no gem so gracious as enterprise." And Doctor Fisk has it. "A dwarf wide awake is now of more use than a giant half asleep; and a living dog is better than a dead lion."

We have only to note the gradation of successful men, to see the world's appreciation of this element. For what else, than to secure the tribute the world ever pays to energy, do we wish to make it appear that we are doing a "driving business?" These things are as they should be.

Nature herself abhors inaction as a vacuum. And I discredit Newton's theory of the *visinertiæ* of matter—its power of having no power—in my greater respect for this other fact of nature.

Now, there are dentists—the Lord have mercy upon them —who are so afflicted with this *visinertiæ*—this "resistance of change as respects motion"—as never to overcome the difficulties of a daily routine of dental practice; they will neither "treat" a dead tooth, nor *extract* it, if it looks any way ominous; they will neither acquire a successful practice, nor practice successfully what they have; I don't know what they calculate upon, but they *do nothing;* they are not hypocrits: but they border clasely on South's ideal hypocrite; "who never opens his mouth in earnest, but when he eats or breaths." Such dentists ever remain in the hot water of beginners; for nothing short of thoroughness, and proficiency, will ever enable one to practice his profession with self-complacency or success.

Sure enough, how shabby and sickly is this starving inaction, be it from what cause it may. Some, like Micawber, are waiting for "something to turn up," while others, like the vulgar herd, are obeying the mandate of some fortuitously successful leader, and "waiting in dignified silence the world to come to them," hoping thereby to secure success, but not so much professionally, as lazily. Fie, fie! Shall not our genius quickly disinherit this imbecility, and break down this unpropitious barrier!

And shall not our profession, in its warfare against quackery, assume the offensive at last, and quickly break it down, or, at least, prevent its devastation of our territory as heretofore? And shall not that part of the profession, assuming the title of it as a whole, acquiese in honorable self-sustaining devisings of genius, and thus render its action more palatable, practical, and profitable to the *majority* whom it would benefit! Now, I am willing to confess to the delicate ground on which I stand here, (or, personally, that only "the wicked stand on slippery places," or any thing else so foreign to the subject,) but I am advocating, for a moment, a reform; and from the very nature of the law of action and reaction, "vice follows on the heels of reformation;"—and yet "the heresy of yesterday is the common-place of to day." Such is the history and experience of all reforms. Why may not our profession expect it, and pass through it, even as our Nation is passing through it to-day. Again, here is not the place for me to justify my position, however easily I could do it. In my suggestions to the youth of the profession I would like to be professional; but if to be successful is to be unprofessional, I profer to run afoul of professionality than miss success,—and for this reason, in the very nature of time and progress professionality *can* and *will* change, while under no circumstances can we hope for a change *in the universal desire for success, and the universal desire to obtain this through the most available channels.* Now, therefore, as I would hope for a professionality or base of action, securing the co-operation of the whole profession, I would adapt it to existing unchangeable circumstances,—and good humoredly enough, feeling that a professionality so framed would ever be in harmony with true ethics, and up with the times, if not with a morbid sensibity. To such as may not see any conflictions between the requirements for success these days in the dental profession, and our professionality, I can but express a regret that I have not the opportunity to point them out; but I may at least drop a suggestion—repeated over and over again on other hands—that there is a great want of unanimity in our profession,—which fact I interpret to mean, a great natural gulf between the professional and unprofessional members of it;—which party alone can move has been intimated.

To return. Having seen to this main item of energy first, we may next see to those "telling virtues, ne'er to be defined"—a thousand little knick-knacks of spicy turn, ever ready to fill their respective chuck holes in our professional career. A man is only to prepare for these as he would for the general emergency; and a complete preparation would not fall short of embracing the whole science of Anthropology. Let his vitality ever keep his wits alive and about him: let him understand how to get his patients, and how retain them; let him know when to be familiar and when formal,—when to smile and when to scorn,—when to extend his hand and when his fist,—when to meet like with like and *vice-versa.* In a word, let him remember that "variety is the spice of life," and carry the thought into his business; and that he can scarcely acquire any fact or accomplishment, that will not tell directly in his favor sometime. A dentist is a fit man for through-culture, extending through the sciences, art and literature. Let him not neglect music. His opportunity for using these in business and society is good.

And again. As much as I am disgusted with all petty combinings of dentistry with any other business' and as much as I could wish to confine this article to success *in* the dental profession, I can not yet refrain from suggesting, that where a dentist has, perchance, a knack for something outside of, and not incompatible with, dentistry; and particularly if his circumstances should demand an extraordinary effort—as in the case of Scott, who at the age ef fifty-six resolutely braced up his energy of mind to discharge a debt of six or seven hundred thousand dollars, by literature; or, if having a thousand hard-earned dollars on hand he would wish to say, like Stephen Gerard, "the first thousand dollars of my immense estates cost me more trouble than all the rest;"—I say, if under such laudable or natural circumstances as these, a dentist may turn an outside-knack greatly to his pecuniary success, I can but suggest that he do it,—and call that genius.

Lastly, and briefly, to refer again to health. Voltaire said, that the fate of nations had often depended on the gout of a prime-minister; and the idea is scarcely an exaggeration of the fact. Health, as an element of success in business or life, can not be ignored. Without it we may have a flashy, effervescent "vim," answering for to-day; but when you come to the requirements of a positive force,—something necessary to keep yourself and family, through a long life, surrounded with the comforts and necessaries pertaining thereto,—without health you will utterly fail. I remember what an advantage a former partner possessed over me in the use of the mouth blow-pipe,—and that I always called it a bull-dog-advantage,—it consisting solely in holding on, and in utter disregard to all blow-pipe theories. I had no little reference to this superior strength in the first of these articles, when I said that success was often purely constitutional. I believe it was Napoleon's idea of providence, that it was always on the strongest side. At any rate, give me my idea of genius,—this energy and health,—and I will overcome all difficulties in the way of success, aye, I will promise to do with the business world what Archimedes did with the natural, if you but gave him a place on which to rest his fulcrum and lever.

I consider it every man's bounden duty, and glorious privilege, to foster health,—and with the dentist, I am surprised that we will so suffer for want of a little precaution. In fact our confinement round our operating chair leaves our vindication from a charge of indolence but little better than that of the two fat noblemen, at the French court, who declared that it was sufficient that each promenaded twice round his friend every morning.

Why will we be eternal complainers and sufferers, and miss success and a "green old age," at last, rather than climb a hill two miles distant every morning before "office hours!" And why deny ourselves the pleasure of four shower baths per week in summer, and two in winter, and wear an undershirt as an apology for our filthiness, or subject our patients to the disgusting odor of our "insensible perspiration," and ourselves to a debasing consciousness, that even inferior animals know nothing of! Surely, these things are *not* as they should be; nor while they exist can we merit a complete uccess.

Piqua, O., Dec., 1862.

